# Emerging roles of astrocytes in hippocampal circuitry and behavior

**DOI:** 10.3389/fncel.2025.1694643

**Published:** 2025-11-21

**Authors:** Ada Squires, Joongkyu Park

**Affiliations:** 1 Department of Pharmacology, Wayne State University School of Medicine, Detroit, MI, United States; 2 Department of Neurology, Wayne State University School of Medicine, Detroit, MI, United States; 3 Translational Neuroscience Program, Wayne State University School of Medicine, Detroit, MI, United States

**Keywords:** astrocyte, hippocampus, calcium signaling, metabolism, synaptic transmission, learning and memory

## Abstract

The discovery of neuronal activity-dependent calcium transients in astrocytes has driven the conceptualization of these cells as computational units in the nervous system. Tripartite synapses, consisting of pre- and postsynaptic terminals along with an adjacent astrocyte, enable astrocytes to communicate with and shape the activity of local synapses. In the hippocampus, astrocytes encode and modulate information through a variety of mechanisms, including tuning the gating of single synapses in their domains, coordinating oscillatory activity across neuronal circuits through astrocytic networks, and providing a foundation for long-term memory formation through intracellular signaling and metabolic coupling. The bidirectional and complementary activities of astrocytes and neurons can be situated in a framework that positions astrocytes as integrators and modulators of neuronal activity, both locally and globally. In this review, we focus on hippocampal astrocytes and discuss recent progress toward understanding astrocytic function in concert with neurons to mediate circuit function and, ultimately, behavior.

## 1 Introduction

The hippocampus plays a crucial role in various brain functions, including learning and memory. By integrating poly-sensory and mnemonic information with cell-intrinsic properties into abstract, plastic representations of behavioral experience, it provides a scaffold for episodic, spatial, and contextual memories. Synaptic plasticity of hippocampal neurons is a critical mechanism by which learning is facilitated (Huganir and Nicoll, 2013; Whitlock et al., 2006). This plasticity, expressed through molecular rearrangements in neuronal compartments, is modulated by the activity of adjacent astrocytes.

Beyond their long-established supporting role, accumulating evidence suggests that astrocytes play an active role in encoding and processing information in the brain. Neuronal activity-dependent calcium signaling in astrocytes has long been observed (Dani et al., 1992), the significance of which has been demonstrated in hippocampus-dependent learning and memory (Adamsky et al., 2018; Kol et al., 2020; Ma et al., 2023; Refaeli et al., 2024; Suthard et al., 2024) and sleep (Foley et al., 2017; Ingiosi et al., 2020; Tsunematsu et al., 2021). Recent investigations have identified diverse forms of hippocampal astrocytic activity in response to synaptic transmission that shape circuit function and behavior, suggesting that these cells are integral for the essential functions of this region (Armbruster et al., 2022; Ioannou et al., 2019; Suzuki et al., 2011; Theparambil et al., 2024). In this review, we discuss recent research that has advanced understanding of the hippocampus by investigating the complementary actions of neurons and astrocytes underlying circuit function and behavior.

## 2 Astrocyte–synapse contacts in domains and across networks

The hippocampus consists of two parallel circuits. The cornu ammonis 1 (CA1) region, the principal output source of the hippocampus, receives inputs directly from the entorhinal cortex (the direct pathway) as well as from the indirect, or trisynaptic, pathway (Basu and Siegelbaum, 2015). The trisynaptic pathway consists of three synaptic connections: first, the perforant pathway links the entorhinal cortex to dentate gyrus (DG) granule cells, which then connect to CA3 pyramidal neurons through the mossy fiber pathway; these in turn synapse onto CA1 pyramidal neurons via the Schaffer collateral (SC) pathway (Basu and Siegelbaum, 2015).

Neuronal terminals are contacted by adjacent astrocytes to form tripartite synapses, which serve to process and transmit information in the hippocampus. Astrocytes have a stellate morphology with fine processes extending from the soma. These processes are highly plastic—for instance, environmental enrichment (Viola et al., 2009), memory encoding (Choi et al., 2016), and aerobic exercise (Saur et al., 2014) enhance the ramification of astrocytic processes in mice. The endfeet of these fine processes ensheathe synapses as perisynaptic astrocytic processes (PAPs) to form tripartite synapses (Aten et al., 2022; Haber et al., 2006) and facilitate bidirectional communication to regulate the structure and activity of astrocytes and neurons. This plasticity supports various hippocampal functions, including learning and memory.

The hippocampus exhibits diverse forms of synaptic plasticity. In addition to the classically established roles of long-term potentiation (LTP) and long-term depression (LTD) in facilitating memory formation (Bliss and Lømo, 1973; Tsien et al., 1996; Whitlock et al., 2006), paradigms such as spike timing-dependent plasticity (STDP) and behavioral timescale plasticity have been developed to further explore the breadth of synaptic plasticity under different physiological and behavioral conditions (Bittner et al., 2017; Magee and Johnston, 1997). LTP occurs at excitatory connections in hippocampal pathways through various mechanisms (Morgan and Teyler, 2001; Zalutsky and Nicoll, 1990). One of the most extensively studied forms depends on *N*-methyl-D-aspartate receptors (NMDARs) and occurs in response to high-frequency stimulation of synapses (Collingridge et al., 1983; Dudek and Bear, 1992; Tsien et al., 1996). NMDARs are ionotropic glutamate receptor complexes that respond to the coincidence of presynaptic glutamate release and postsynaptic depolarization with calcium influx at the postsynaptic membrane. This activates a calcium/calmodulin-dependent protein kinase II-dependent pathway by which α-amino-3-hydroxy-5-methyl-4-isoxazolepropionic acid receptors (AMPARs)—another class of ionotropic glutamate receptors—are trafficked to the postsynaptic membrane, thereby augmenting synaptic strength (Hayashi et al., 2000; Malenka et al., 1989; Malinow et al., 1989). LTD, in contrast, occurs in response to low-frequency stimulation of a synapse; NMDAR-dependent LTD mediates the endocytosis of AMPARs, thereby diminishing synaptic strength (Beattie et al., 2000; Dudek and Bear, 1992; Mulkey et al., 1994). This process is critical for a variety of hippocampal processes, including novelty acquisition (Dong et al., 2012; Kemp and Manahan-Vaughan, 2004; Kim et al., 2011; Manahan-Vaughan and Braunewell, 1999) and cognitive flexibility (Nicholls et al., 2008).

The established view of synaptic plasticity has been expanded by evidence suggesting a role for astrocytes in modulating neuronal activity to shape hippocampal function. Across brain regions, astrocytes support neuronal function through a variety of mechanisms, including the buffering of ions (Bellot-Saez et al., 2017; Nowak et al., 1987; Rose and Ransom, 1996), uptake of neurotransmitters (Bergles and Jahr, 1997; Gadea and López-Colomé, 2001), coordination of metabolism (Rouach et al., 2008), and regulation of synaptogenesis (Christopherson et al., 2005; Eroglu et al., 2009). Beyond these homeostatic functions, astrocytes are critical in facilitating the transmission of information through neuronal circuits. PAPs, as the astrocytic component of the tripartite synapse, are regulated by neuronal activity, both morphologically (Bernardinelli et al., 2014; Cornell-Bell et al., 1990) and translationally (Sapkota et al., 2022). In turn, astrocytes can phagocytose synapses in the CA1 region in response to neuronal activity throughout adulthood, a process critical to sustaining LTP and memory formation by eliminating superfluous excitatory connections (Lee et al., 2021). These changes are highly circuit-dependent; accordingly, distinct astrocytic transcriptional programs emerge across different brain regions during development to govern the circuit specificity of tripartite synapses (Huang et al., 2020).

Astrocytes are involved in modulating various forms of synaptic plasticity. STDP modulates the strength of a synapse based on the temporal dynamics of pre- and postsynaptic activity (Magee and Johnston, 1997). If a presynaptic neuron fires a short time before the postsynaptic neuron does, the synapse strengthens; by contrast, if postsynaptic activity precedes presynaptic activity, the synapse weakens. During postnatal development, SC–CA1 synapses transition from spike timing-dependent LTD to spike timing-dependent LTP, a process dependent on downregulating NMDAR expression and upregulating metabotropic glutamate receptor (mGluR) expression, as well as astrocytic calcium activity and glutamate release (Falcón-Moya et al., 2020). This astrocytic activity and subsequent vesicular release are also critical for the induction of spike timing-dependent LTD at perforant pathway–DG synapses (Martínez-Gallego et al., 2024).

**FIGURE 1:**
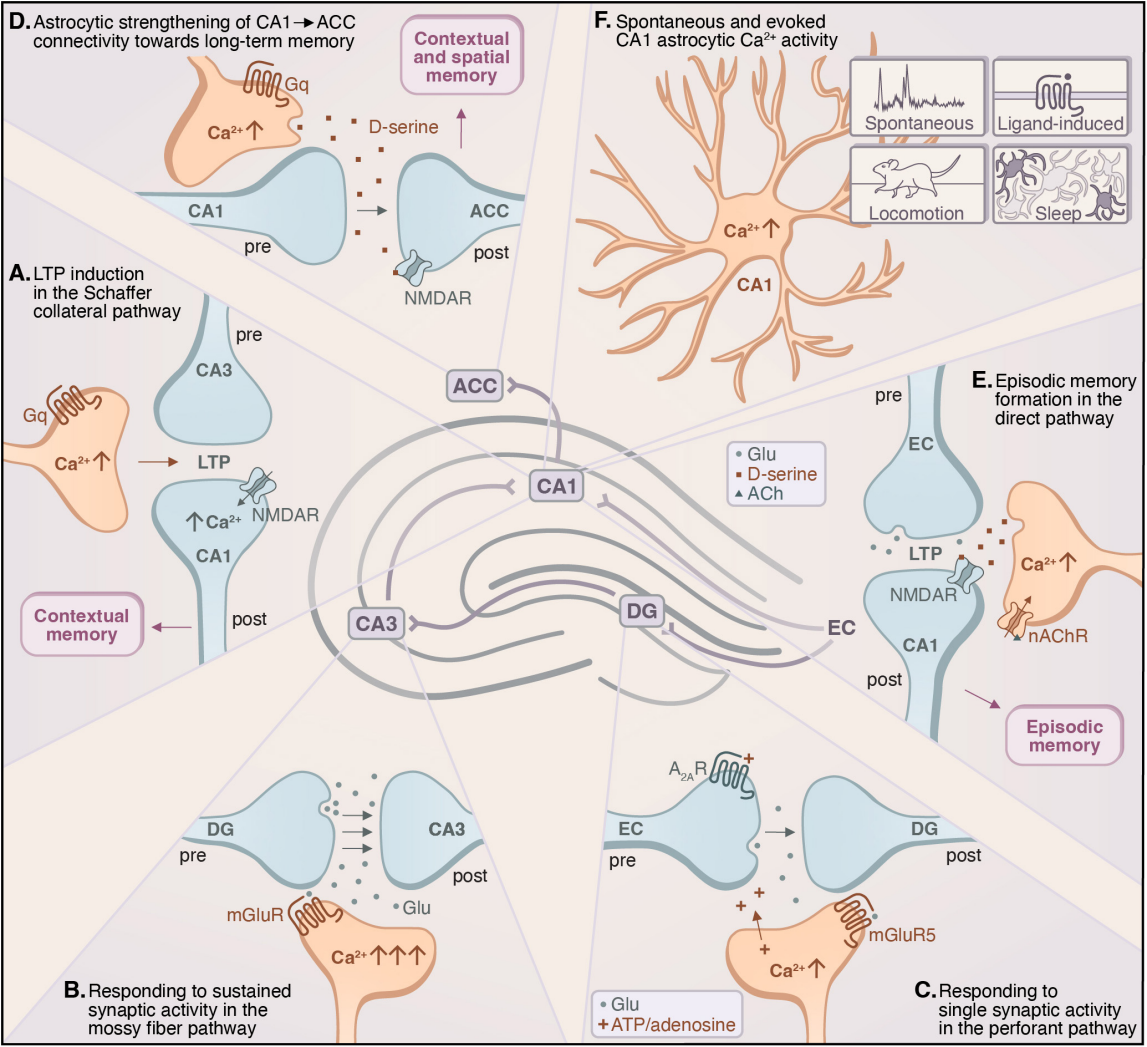
Hippocampal astrocytes exhibit diverse forms of calcium signaling in response to neuronal activity, both in the hippocampus and in connections to and from the hippocampus, to shape behavior. **(A)** Astrocytic activation in the Schaffer collateral (SC)–CA1 pathway induces LTP through the activation of postsynaptic NMDARs (Adamsky et al., 2018). **(B)** Sustained glutamatergic activity in the mossy fiber–CA3 pathway elicits broad calcium activity in nearby astrocytes (Haustein et al., 2014). **(C)** Single synaptic activity in the perforant pathway–dentate gyrus (DG) elicits calcium transients in adjacent astrocytes and potentiates synapses through the astrocytic release of ATP/adenosine (Di Castro et al., 2011). **(D)** CA1 astrocytic activation promotes functional connectivity between the CA1 region and the anterior cingulate cortex (ACC) to promote long-term contextual and spatial memory (Adamsky et al., 2018; Kol et al., 2020; Refaeli et al., 2024). **(E)** The activation of CA1 astrocytic calcium signaling promotes the induction of LTP in the direct pathway–CA1 by eliciting D-serine release downstream of nicotinic cholinergic activity, facilitating the formation of episodic memory by modulating neuronal NMDAR activity (Ma et al., 2023). **(F)** Calcium activity in the CA1 astrocytes can be elicited spontaneously or by the binding of a ligand (often a neurotransmitter or neuromodulator) to an astrocytic receptor, and is modulated by locomotion and sleep/wakefulness (Bindocci et al., 2017; Dani et al., 1992; Doron et al., 2022; Duffy and MacVicar, 1995; Haustein et al., 2014; Ingiosi et al., 2020; Nett et al., 2002; Rupprecht et al., 2024; Sharma and Vijayaraghavan, 2001; Tang et al., 2015; Tsunematsu et al., 2021; Wu et al., 2014).

**FIGURE 2:**
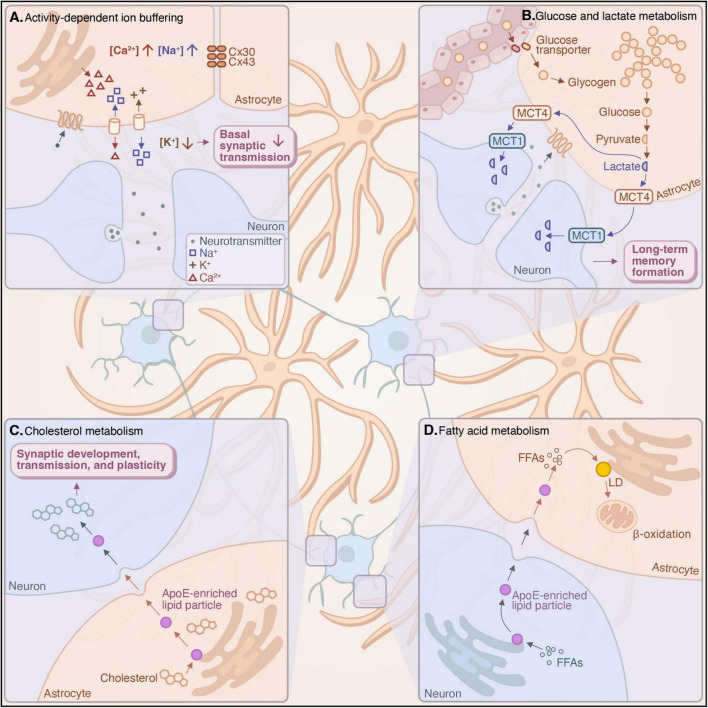
Hippocampal astrocytes form networks through gap-junction coupling, which play a crucial role in buffering ions and neurotransmitters and facilitating metabolic coupling with neurons. **(A)** Astrocytic networks mediate the uptake and release of ions to buffer extracellular potassium concentrations, thereby modulating synaptic transmission and enhancing synaptic fidelity (Chever et al., 2016; Dossi et al., 2024; Wang et al., 2012; Zhong et al., 2023). **(B)** Astrocytes shuttle glucose across the blood-brain barrier (BBB) and produce lactate through aerobic glycolysis, the delivery of which to hippocampal neurons is critical for the formation of long-term memory (Gao et al., 2016; Suzuki et al., 2011). **(C)** Astrocytic cholesterol biosynthesis and its delivery to neurons via lipoprotein particles are necessary for synaptic development, transmission, and plasticity (Ferris et al., 2017; Fünfschilling et al., 2007; van Deijk et al., 2017). **(D)** Astrocytes take up fatty acids from neurons in response to neuronal activation via ApoE-enriched particles for storage in lipid droplets (LDs), which can subsequently be mobilized to provide energy to neurons through β-oxidation (Chen et al., 2023; Ioannou et al., 2019; Qi et al., 2021).

As a paradigm, it may be useful to consider two complementary scopes by which astrocytes regulate synaptic function. First, astrocytes form structural and functional domains that tile the central nervous system, intermingling with the fine processes of adjacent astrocytes only at the periphery of the cell (Grosche et al., 2013; Ogata and Kosaka, 2002; Refaeli et al., 2021; Viana et al., 2023). This potentially allows the modular regulation of synapses in the domain of an astrocyte (Figure 1). Second, astrocytes form networks through gap junction coupling, which mediates the broad coordination of neuronal circuits as well as the intercellular flow of nutrients to sustain synaptic transmission (Figure 2). Both astrocytic domains and networks emerge early in postnatal development (Bushong et al., 2004; Zhong et al., 2023), and their functions are critical for learning and memory in the hippocampus (Rouach et al., 2008; Suzuki et al., 2011). These two perspectives of astrocytic function are not mutually exclusive but are often interwoven by molecular crosstalk between neurons and astrocytes (Bonvento and Bolaños, 2021). By facilitating both the discretized and integrated regulation of synaptic transmission, astrocytes are able to dynamically and precisely modulate hippocampal circuitry.

## 3 Astrocytic signaling during behavior

Investigations into hippocampal astrocytic activity, particularly calcium activity, have been demonstrated to be important for memory processing. The initial discovery that glutamate elicits transient changes in intracellular calcium concentrations in hippocampal astrocytes (Dani et al., 1992) spurred the conceptualization of these cells as actively encoding and transmitting information. Indeed, later investigations demonstrated that astrocytic intracellular calcium activity, when coincident with neuronal activation, is sufficient to induce LTP at single hippocampal synapses and facilitate memory formation (Adamsky et al., 2018; Perea and Araque, 2007; Figure 1A).

Much of the research to date has examined astrocytic signaling through calcium dynamics. While other forms of neuronal activity-dependent signaling have been demonstrated in astrocytes (Armbruster et al., 2022; Theparambil et al., 2024), their behavioral significance is still emerging. For instance, genetically encoded voltage indicators have revealed changes in astrocytic membrane potential during neuronal activity. Microdomain depolarizations in PAPs occur during synaptic activity, mediated by potassium uptake as well as the electrogenic uptake of glutamate (Armbruster et al., 2022). This cellular response to neuronal activity suggests that molecular mechanisms beyond those of calcium may contribute to astrocytic encoding and regulation of synaptic transmission. Nevertheless, investigations into astrocytic calcium dynamics have revealed their profound role in shaping hippocampal circuit activity.

Advances in molecular tools, such as the development of genetically encoded calcium indicators (e.g., GCaMP), have allowed investigators to finely elucidate calcium activity in astrocytes at rest and in response to neuronal activity. This cellular activity has been demonstrated to be indispensable for mediating information flow across synapses and for shaping hippocampus-dependent behavior. Investigating the dynamics and significance of astrocytic calcium signaling in response to neuronal activity will continue to provide a more comprehensive understanding of hippocampal function.

### 3.1 Calcium dynamics in astrocytes

Hippocampal astrocytes exhibit distinct spatiotemporal calcium activity both at rest and in response to synaptic transmission. At rest, intracellular calcium concentrations in astrocytes are unevenly distributed throughout the cell (Zheng et al., 2015), influencing the peak level and amplitude of calcium signals (King et al., 2020). This heterogeneity suggests the existence of functional microdomains in astrocytes, through which these cells may modulate the activity of individual synapses. Astrocytes can respond to synaptic transmission with calcium activity but also exhibit spontaneous calcium transients independent of neuronal activity (Haustein et al., 2014; Nett et al., 2002). Evoked astrocytic calcium activity can arise from either extracellular or intracellular sources, such as the endoplasmic reticulum through inositol trisphosphate (IP_3_) signaling (Agarwal et al., 2017; Glaum et al., 1990; Perea and Araque, 2005). By contrast, spontaneous astrocytic activity occurs independently of IP_3_ signaling (Srinivasan et al., 2015), instead relying on the influx of extracellular calcium (Hjukse et al., 2023; Rungta et al., 2016) and calcium efflux from mitochondria (Agarwal et al., 2017). This spontaneous activity exhibits distinct temporal dynamics, in which the duration of a signal is generally logarithmically proportional to its spatial distribution (Wu et al., 2014). In the hippocampus, PAPs form calcium microdomains; that is, astrocytic fine processes apposing synapses exhibit distinct calcium signaling generally restricted to the region in which it originated (Arizono et al., 2020). This suggests that a single astrocyte may be structurally and functionally capable of differentially communicating with many synapses in parallel in its domain.

Calcium activity in an astrocyte varies depending on the synapses in its domain. For instance, in the mossy fiber–CA3 pathway, sustained glutamate release elicits broad calcium activity throughout surrounding astrocytes, dependent on mGluR activation (Haustein et al., 2014; Figure 1B). By contrast, astrocytes in the perforant pathway–DG (Di Castro et al., 2011) and SC–CA1 pathway can respond to single synaptic activity with calcium transients, a process dependent on astrocytic mGluR5 and purinergic signaling (Bernardinelli et al., 2011; Panatier et al., 2011; Figure 1C). Astrocytes in the perforant pathway–DG can also encode coincident transmission across synapses through IP_3_-mediated calcium activity (Di Castro et al., 2011). Moreover, calcium activity in hippocampal astrocytes is modulated by behavior. For instance, locomotion tends to be accompanied by neuronal signaling from the locus coeruleus, which activates α1-adrenergic receptors on CA1 astrocytes to trigger broad, synchronized calcium events in hippocampal astrocytes, during which calcium transients originating from fine processes intensify and propagate toward the soma simultaneously across a population of astrocytes (Bindocci et al., 2017; Rupprecht et al., 2024). This circuit- and behavior-dependent signaling suggests a role for astrocytes in encoding and integrating synaptic transmission. The specific effectors upstream of this signaling that confer the consistent calcium signatures often observed in response to neuronal activity, as well as the downstream effectors that promote its behavioral effects, remain open questions. Nevertheless, experimental manipulation of astrocytic calcium signaling has provided insights into its behavioral relevance, highlighting its essential roles in hippocampal function.

### 3.2 Learning and memory

The use of chemogenetic and optogenetic approaches to activate astrocytes *in vivo* has provided valuable insights into the role of these cells in mediating hippocampus-dependent memory. The hippocampus plays an important role in processing declarative, or explicit, memory (Squire, 2004), spatial information and memory (O’Keefe and Burgess, 1996; Tsien et al., 1996), and episodic memory (Kraus et al., 2013; MacDonald et al., 2013; Pastalkova et al., 2008). Both neurons and astrocytes in the ventral CA1 region exhibit distinct patterns of calcium activity during memory acquisition. Optogenetic reactivation of this population of DG neurons elicits calcium activity in both cell types in the CA1 region resembling that of natural recall, concomitant with the artificial recall of the memory (Suthard et al., 2024). The functional significance of this signaling in CA1 astrocytes has been elucidated through genetic manipulations in these cells, which demonstrate its sufficiency in facilitating the long-term consolidation of memory by promoting cortico-hippocampal synaptic connectivity (Adamsky et al., 2018; Kol et al., 2020; Refaeli et al., 2024). Calcium signaling occurs in astrocytes, at least in part, through G-protein-coupled receptors (GPCRs), including mGluRs, γ-aminobutyric acid B (GABA_*B*_) receptors, adenosine receptors, adrenergic receptors, and cannabinoid receptors, which elicit the intracellular release of calcium from the endoplasmic reticulum through IP_3_ signaling (Agulhon et al., 2010; Araque et al., 2002; Coiret et al., 2012; Duffy and MacVicar, 1995; Panatier et al., 2011; Pascual et al., 2005; Petravicz et al., 2008; Serrano et al., 2006). In neurons, GPCRs coupled to Gq proteins elicit intracellular calcium activity, while GPCRs coupled to Gi proteins diminish this activity. By contrast, experimental stimulation of both Gq and Gi signaling activates calcium signaling in astrocytes (Durkee et al., 2019). These forms of G protein signaling have differing effects in different cell types, as CA1 astrocytic Gq signaling enhances the recall of hippocampus-dependent memory, while neuronal Gq signaling and astrocytic Gi signaling impair this recall (Adamsky et al., 2018; Kol et al., 2020; Refaeli et al., 2024).

A robust paradigm for studying hippocampus-dependent memory is contextual fear memory, wherein an animal’s fear response to a spatial environment previously coupled with an aversive stimulus is recorded as a correlate of memory. Chemogenetic stimulation of CA1 astrocytic Gq signaling during fear memory acquisition enhances recent recall of contextual memory in mice by eliciting the release of D-serine, activating neuronal NMDARs, and strengthening functional connectivity between the CA1 region and anterior cingulate cortex (ACC) (Adamsky et al., 2018; Refaeli et al., 2024; Figure 1D). By contrast, chemogenetic Gi-GPCR activation in CA1 astrocytes during memory acquisition impairs remote memory recall, concomitant with reduced functional connectivity between CA1 and ACC (Kol et al., 2020; Refaeli et al., 2024). These findings suggest that the Gq pathway in hippocampal astrocytes facilitates memory consolidation by strengthening the synaptic connections between CA1 and ACC, while the Gi pathway exerts the opposite effect, thereby diminishing recall. The temporal differences between the effects of Gq and Gi signaling may be due to the progressive increase in CA1–ACC functional connectivity during memory consolidation; therefore, Gq activation has the greatest impact at an earlier time point, when connectivity is generally lower, and Gi activation has the greatest impact at a later time point, when connectivity is generally higher (Refaeli et al., 2024). Overall, these results suggest that CA1 astrocytes play a crucial role in the long-term consolidation of memories by mediating the transfer of hippocampus-dependent memories into cortical areas.

Astrocytes play a role in regulating memory engrams in the hippocampus. Engrams have been proposed as the structural and functional changes in neuronal ensembles associated with memory, serving as the cellular substrate of memory (Josselyn et al., 2017). In the hippocampus, memory acquisition is considered the strengthening of synaptic connections among a constellation of neurons. After memory consolidation, their reactivation—induced either artificially or through the natural retrieval of the memory—reliably elicits recall of the memory from which they were formed (Liu et al., 2012; Ramirez et al., 2013). Engram neurons are often identified by their upregulated expression of immediate early genes (e.g., *c-Fos*) shortly after memory acquisition (Liu et al., 2012). Intriguingly, learning also induces *c-Fos* expression in a subset of astrocytes in the CA1 and DG. Engram neurons are located in the domains of these *c-Fos*-expressing astrocytes (Williamson et al., 2025). Astrocyte-specific knockout of this expression reduces hippocampal LTP and contextual memory formation (Williamson et al., 2025), while chemogenetic activation of these peri-engram astrocytes promotes LTP at SC–CA1 synapses and evokes the artificial recall of a contextual memory outside of the context in which it was formed, suggesting that this subset of hippocampal astrocytes is specifically crucial for mediating memory recall (Williamson et al., 2025).

Hippocampal astrocytic calcium activity also contributes to the acquisition and maintenance of spatial memory, as chemogenetic activation of Gq-GPCR signaling in CA1 astrocytes enhances spatial memory in mice (Adamsky et al., 2018). The discovery of place cells in the hippocampus, which exhibit activity when the animal is in a certain location (O’Keefe and Dostrovsky, 1971), was a pivotal breakthrough in understanding how this region encodes spatial information. A population of place cells, known as a place field, forms an abstract representation of space in the hippocampus (Kjelstrup et al., 2008). The activity of these neurons can be decoded to determine the location of an animal moving through a familiar environment. While the classical view of place fields in the hippocampus involves only neurons, calcium imaging in freely behaving mice has identified topographically organized astrocyte activity in the hippocampus, to the extent that decoding the location of the mouse is more accurate when astrocytic signals are considered alongside neuronal ones (Curreli et al., 2022). Furthermore, in CA1 astrocytes, intracellular calcium activity increases as a mouse approaches a reward (Doron et al., 2022), suggesting that spatially dependent activity in hippocampal astrocytes may aid in recollecting a behaviorally salient feature of the environment. However, this calcium activity could also reflect past events, such as changes in neuronal activity, locomotion, or pupil dilation (Rupprecht et al., 2024).

Astrocytic activity has also been shown to be critical for episodic memory formation (Fortin et al., 2002). Time cells in the CA1 region are a critical feature of hippocampal temporal encoding (Kraus et al., 2013; MacDonald et al., 2011; MacDonald et al., 2013). These cells exhibit temporally specific firing patterns, analogous to the spatial specificity of place cells. In fact, the same population of CA1 neurons can function as either time cells or place cells (Cabral et al., 2014; Kraus et al., 2013). These neurons replay their spatial or temporal sequences during quiet wake to facilitate consolidation (Carr et al., 2011). CA1 astrocytes appear to contribute to episodic memory: chemogenetic or optogenetic activation of CA1 astrocytes enhances LTP at synapses in the direct pathway–CA1 and promotes episodic memory formation, while disruption of this signaling through astrocyte-specific expression of a plasma membrane calcium pump (hPMCA2w/b) diminishes this form of memory (Ma et al., 2023; Figure 1E).

Overall, astrocytic calcium signaling in the hippocampus, particularly in the CA1 region, has been demonstrated to be sufficient for coordinating neuronal activity toward the formation and long-term consolidation of hippocampus-dependent memory, including contextual, spatial, and episodic memory. This calcium activity occurs both spontaneously and in response to neuronal or behavioral changes (Figure 1F), suggesting a capacity for CA1 astrocytes to dynamically tune hippocampal neuronal activity to the context from which it arises. However, fundamental questions remain, such as the potential role of astrocytes in allocating neurons to an engram, as well as the extent to which astrocytes contribute to the spatiotemporal properties of behavioral experience through their proximity to place and time cells. Exploring these questions will further elucidate the cellular mechanisms underlying memory, as well as the emergent properties of experience constructed by the hippocampus in relation to learning and memory.

### 3.3 Sleep and wakefulness

Sleep is critical for maintaining hippocampal function and promoting memory consolidation (Karni et al., 1994; Walker and Stickgold, 2004). The four stages of sleep include three non-rapid eye movement (NREM) stages and one rapid eye movement (REM) stage. Stage 3 of NREM sleep, also known as slow-wave sleep, is characterized by neuronal delta (0.5–4 Hz) oscillations. During slow-wave sleep, hippocampal neuronal ensembles are reactivated to promote memory consolidation (Lee and Wilson, 2002; Wilson and McNaughton, 1994). During REM sleep, cholinergic projections to the hippocampus mediate theta (4–7 Hz) oscillations, which are also crucial for memory consolidation (Griffin et al., 2004; Marrosu et al., 1995).

The activities of neurons and astrocytes vary across sleep stages. Hippocampal synaptic transmission becomes synchronized during sleep, reaching its peak during NREM sleep and exhibiting its lowest levels of synchronization during wakefulness (Bódizs et al., 2001; Moroni et al., 2007). By contrast, astrocytic calcium signaling desynchronizes during NREM sleep and synchronizes during wakefulness, a state in which increased vigilance promotes broad, coordinated calcium activity across astrocytes (Bindocci et al., 2017; Ingiosi et al., 2020; Rupprecht et al., 2024; Tsunematsu et al., 2021). Calcium concentrations in hippocampal astrocytes are highest during wakefulness and lowest during REM sleep, a phenomenon reliable enough that astrocytic calcium activity in the hippocampus can be decoded to predict sleep and wakefulness states (Tsunematsu et al., 2021).

Furthermore, the manipulation of calcium activity in hippocampal astrocytes has revealed their role in mediating sleep behavior. A study in which astrocytic calcium signaling was experimentally diminished through knockout of stromal interaction molecule 1 revealed its role in regulating sleep; this manipulation reduced both sleep time and sleep pressure in mice (Ingiosi et al., 2020). In another study, mice with disrupted astrocytic calcium signaling, achieved by expression of IP_3_ 5’-phosphatase, entered REM sleep more frequently, concomitant with increased hippocampal theta oscillations (Foley et al., 2017). Together, these studies suggest a role for hippocampal astrocytic calcium signaling in sleep homeostasis.

### 3.4 Tripartite synapses in neurodegeneration

Investigations of astrocytes in various mouse models of Alzheimer’s disease (AD) have revealed profound changes in neuron–astrocyte interactions in the hippocampus that mediate the pathogenesis of neurodegeneration. AD is a progressive neurodegenerative disorder characterized by the deposition of β-amyloid (Aβ) plaques and neurofibrillary tangles containing hyperphosphorylated tau (Long and Holtzman, 2019).

Various AD mouse models have revealed changes in tripartite synaptic structure and function that may contribute to AD pathologies. The APP/PS1 mouse model, which carries a mutant amyloid precursor protein (APP) and a mutant presenilin 1 (PS1), exhibits Aβ pathology, gliosis, and memory deficits by 6 months of age (Jankowsky et al., 2004). Transcriptomic studies have revealed that APP/PS1 astrocytes in the hippocampus are deficient in genes critical for synaptic function and neurotransmitter homeostasis (Jiwaji et al., 2022; Endo et al., 2022). Furthermore, APP/PS1 astrocytes in the hippocampus aberrantly phagocytose synapses for lysosomal degradation (Li et al., 2024; St-Pierre et al., 2023) and exhibit deficits in their support of the axon initial segment early in development (Benitez et al., 2024). These changes may contribute to neurodegeneration by compromising the integrity of hippocampal tripartite synapses and reducing their capacity for learning-induced plasticity. For instance, the structural changes observed in tripartite synapses after memory acquisition, dependent on morphological plasticity of PAPs, are altered in APP/PS1 mice, concomitant with impaired memory formation (Kater et al., 2023).

Neuronal hyperactivity has been considered a crucial event in the pathogenesis of AD, which contributes to aberrant neural network changes and excitotoxicity. Both Aβ and tau pathology can contribute to changes in astrocyte–neuron coupling that promote hyperactivity. For instance, tau pathology downregulates glutamate transporter 1 expression in astrocytes, which diminishes the capacity of astrocytes to buffer this excitatory neurotransmitter (Puma et al., 2022). Changes in astrocytic calcium activity, downstream of Aβ pathology, may be a triggering event for neuronal hyperactivity in the hippocampus in neurodegenerative contexts. Aβ production upregulates mGluR5 and IP_3_ receptor expression in hippocampal astrocytes (Grolla et al., 2013a; Grolla et al., 2013b; Ronco et al., 2014). This upregulated expression elicits calcium hyperactivity in astrocytes (Ronco et al., 2014). Aβ-induced calcium hyperactivity in astrocytes, which is observed in multiple mouse models of AD (Huffels et al., 2022; Ronco et al., 2014), can increase glutamatergic neuronal activity in the CA1 region (Bosson et al., 2017), which is dependent on astrocytic calcium-dependent glutamate release and neuronal NMDAR activation (Paumier et al., 2022; Pirttimaki et al., 2013), and trigger synaptic spine loss (Talantova et al., 2013). This increased spontaneous astrocytic calcium activity is correlated with vascular tone instability, which promotes neuronal death (Takano et al., 2007). Furthermore, high concentrations of calcium in the astrocytic endoplasmic reticulum may contribute to endoplasmic reticulum stress as well as a reduction in protein synthesis (Dematteis et al., 2020; Dematteis et al., 2025; Tapella et al., 2022). This cellular stress induces aberrant changes in mitochondrial morphology and increases reactive oxygen species production from astrocytes. Metabolic adaptations downstream of these changes increase the cellular use of glutamine, which elicits the excessive release of glutamate (Carvalho et al., 2023), further contributing to neuronal hyperactivity.

Overall, astrocytic calcium signaling plays a crucial role in facilitating information flow and mediating both the homeostatic and behavioral functions of the hippocampus in health and disease. Astrocytes can modulate the activity of synapses in their domains through precise and localized signaling in their fine processes. This discretized, specific modulation of information flow is complemented by the activity of extensive networks formed by adjacent astrocytes that allow the regulation of neuronal circuits. Both levels of astrocytic organization are indispensable for hippocampal function, positioning astrocytes to play an active role in processing information both locally and broadly.

## 4 Neuron–astrocyte metabolic coupling and astrocytic networks

Interconnected networks of astrocytes play an important role in buffering ions and shuttling metabolites to shape neuronal activity. These networks enable neuron–astrocyte electrical coupling through the buffering of ions and neurotransmitters (Dossi et al., 2024; Zhong et al., 2023), which is critical for maintaining basal synaptic transmission (Wang et al., 2012) as well as tuning the signal-to-noise ratio of synaptic transmission to promote the synchronization of neuronal ensembles (Chever et al., 2016). They also facilitate the intercellular trafficking of nutrients for neuronal uptake after their transport across the blood-brain barrier (BBB), a structure formed by contacts between astrocytic endfeet and vascular endothelial cells (Abbott, 2002; Nortley and Attwell, 2017). This metabolic coupling between neurons and astrocytes is necessary to deliver essential metabolites to hippocampal neurons (Rouach et al., 2008), thereby supporting long-term memory formation (Suzuki et al., 2011).

Astrocytic networks are formed through gap junction coupling among adjacent astrocytes. The gap junction proteins that form these networks are connexin-30 and connexin-43 (Dermietzel, 1974; Masa and Mugnaini, 1982). Memory consolidation upregulates connexin-43 expression in the hippocampus (Choi et al., 2016), suggesting that these networks dynamically respond to neuronal activity and behavioral changes. Conditional knockout of one or both of these gap junction proteins specifically in astrocytes has revealed their role in maintaining basal synaptic transmission and facilitating learning and memory. Loss of gap junction coupling leads to extracellular accumulation of potassium ions and glutamate and abolishes the activity-dependent shuttling of glucose and its metabolites through astrocytic networks. These knockout models show invasion of astrocytic processes into synaptic clefts, alterations in basal synaptic transmission and LTP, and a reduction in memory formation (Hösli et al., 2022; Pannasch et al., 2014; Rouach et al., 2008; Theis et al., 2003; Wallraff et al., 2006). These results highlight the necessity of astrocytic networks in buffering ions and neurotransmitters and in trafficking metabolites to ensure the fidelity and metabolic support of hippocampal synaptic transmission toward learning and memory.

The development of astrocytic networks coincides with the coordinated buffering of sodium and potassium ions by astrocytes and neurons that characterizes syncytial isopotentiality (Zhong et al., 2023). These networks regulate neuronal synchronization and oscillations by modulating the balance between excitatory synaptic transmission and release probability. Generally, there is an inverse relationship between basal excitatory activity and synaptic release probability, a relationship that can be likened to a signal-to-noise ratio of synaptic transmission. Astrocytic networks help maintain this balance (Wang et al., 2012), promoting neuronal synchronization by reducing basal synaptic activity and thereby enhancing release probability (Chever et al., 2016). Mechanistically, this may be achieved through the buffering of extracellular potassium (Dossi et al., 2024). Together, these findings suggest that astrocytic networks are critical for maintaining and modulating the electrochemical gradient across neuronal membranes to shape the basal activity of neuronal circuits (Figure 2A).

Astrocytic networks also play a crucial role in brain metabolism. Metabolic coordination between neurons and astrocytes is essential to brain function. The coupling of glucose and lipid metabolism between these cell types allows astrocytes to sustain synaptic transmission and promote long-term memory formation through the shuttling of glucose and its metabolites, the buffering of potentially toxic lipid species generated by neuronal activity, and the provision of cholesterol to support synaptic plasticity (Ferris et al., 2017; Ioannou et al., 2019; Rouach et al., 2008; Suzuki et al., 2011). These neuronal activity-dependent metabolic functions are especially critical in the hippocampus; for instance, the delivery of glucose and its metabolites by astrocytic networks sustains neuronal synchronization and epileptiform events (Rouach et al., 2008), the transfer of the glycolytic metabolite lactate from hippocampal astrocytes to neurons is required for the formation of long-term memories (Gao et al., 2016; Suzuki et al., 2011), and defects in hippocampal astrocytic lipid metabolism are implicated in neurodegeneration (Qi et al., 2021).

### 4.1 Glucose metabolism and memory

Astrocytes are responsible for the uptake and transport of glucose and its metabolites from central circulation to neurons throughout the brain. Perivascular astrocytes contact blood vessels through their endfeet to form the BBB. These astrocytes are positioned for glucose uptake, which can subsequently be metabolized and trafficked intercellularly through astrocytic networks. Glucose transporters in astrocytic foot processes shuttle glucose across the BBB (Koepsell, 2020; Morgello et al., 1995), whereupon it can be released for neuronal uptake, transported through astrocytic networks to meet the energetic needs of distal neurons (Rouach et al., 2008), or stored intracellularly as glycogen (Figure 2B). In transit, glucose may be metabolized through aerobic glycolysis, the oxygen-dependent conversion of glucose into lactate. This mechanism depends on oxygen availability as well as astrocytic calcium concentration, which in turn regulates vasodilation (Gordon et al., 2008), indicating the intricacy with which astrocytes coordinate metabolism across the BBB. Glycolytic pathways can also metabolize glucose into L-serine, the precursor of D-serine, a critical transmitter released by astrocytes to regulate neuronal activity (Henneberger et al., 2010; Suzuki et al., 2015).

The astrocyte–neuron lactate shuttle hypothesis suggests that astrocytes take up glutamate in response to neuronal activity, which in turn stimulates aerobic glycolysis to sustain synaptic activity through the provision of lactate to neurons (Pellerin and Magistretti, 1994). While studies have suggested that this metabolic coupling underlies synaptic activity (Bittner et al., 2011; Pellerin and Magistretti, 1994; Pellerin et al., 2007), other evidence indicates that sustained neuronal activity relies on neuronal glycolysis rather than lactate uptake, as neurons exhibit a net export of lactate under stimulation (Díaz-García et al., 2017). Although the precise role of coordinated lactate metabolism among astrocytes and neurons in supporting basal synaptic activity remains unresolved, the transport of glucose and its metabolites through astrocytic networks in response to neuronal activity appears to be necessary to locally support synaptic transmission in the hippocampus (Rouach et al., 2008).

Beyond basal activity, evidence suggests that astrocyte–neuron lactate metabolism is critical for LTP and memory formation (Gao et al., 2016; Suzuki et al., 2011; Figure 2B). Astrocytic aerobic glycolysis can be elicited by neuronal activity, such as the release of glutamate (Pellerin and Magistretti, 1994), norepinephrine (Gao et al., 2016), or adenosine (Theparambil et al., 2024), promoting the transport of lactate from astrocytes to neurons to support LTP. The breakdown of astrocytic glycogen into lactate through glycogenolysis and its transport via monocarboxylate transporters (MCTs) for neuronal uptake is required for long-term memory formation in rats (Suzuki et al., 2011). MCT1 is primarily expressed by neurons, while MCT4 is enriched in astrocytes (Pierre and Pellerin, 2005). The expression of MCT1 is upregulated during memory encoding (Suzuki et al., 2011), indicating an increased capacity for neuronal lactate uptake. MCT1- or MCT4-deficient rodents exhibit impaired memory of aversive stimuli (Suzuki et al., 2011; Tadi et al., 2015), which is rescued by lactate administration when neuronal uptake is not disrupted (Suzuki et al., 2011). Furthermore, AD mouse models exhibit defects in hippocampal astrocytic glycolysis, which is restored by upregulating astrocytic glycolysis or administering glycolytic metabolites (Andersen et al., 2021; Dematteis et al., 2020; Le Douce et al., 2020; Minhas et al., 2024). This suggests that astrocytic glucose metabolism and the provision of lactate from astrocytes to neurons are specifically necessary for the formation of hippocampus-dependent long-term memory. Whether this lactate transport simply supports neurons metabolically or has an additional signaling function in the hippocampus remains to be determined.

### 4.2 Lipid metabolism and neurodegeneration

Astrocytes are critical in mediating brain cholesterol metabolism (Figure 2C). Developing neurons are especially rich in cholesterol (Zhang and Liu, 2015), which is essential for synaptogenesis (Fester et al., 2009; Goritz et al., 2005; Mauch et al., 2001), axonal guidance (de Chaves et al., 1997), and synaptic transmission (Linetti et al., 2010; Liu et al., 2010). Because it cannot cross the BBB, cholesterol is synthesized *de novo* in the brain, primarily in astrocytes by adulthood (Ferris et al., 2017; Fünfschilling et al., 2007; van Deijk et al., 2017). Disrupting astrocytic cholesterol biosynthesis results in significant reductions in hippocampal volume and deficits in spatial memory in wild-type mice and aggravates Aβ pathology in AD mice (Ferris et al., 2017; Wang et al., 2021). APP/PS1 mice also display disrupted cholesterol homeostasis in hippocampal astrocytes (Endo et al., 2022; Habib et al., 2020), suggesting that this metabolic coupling is essential for both the homeostatic and behavioral functions of the hippocampus.

Apolipoprotein E (ApoE), a principal cholesterol carrier, is expressed by astrocytes to facilitate cholesterol delivery to neurons (Wang and Eckel, 2014), where it plays a critical role in synapse formation, plasticity, and transmission (Hayashi et al., 2004; Holtzman et al., 1995; Mauch et al., 2001). Astrocytic ApoE also regulates cholesterol metabolism and epigenetic modifications in hippocampal neurons to facilitate memory formation (Li et al., 2021). ApoE4 expression in astrocytes disrupts cholesterol metabolism, resulting in reduced astrocytic cholesterol efflux. This suggests that the provision of cholesterol from astrocytes to neurons throughout life is essential to their function, and that deficits in astrocytic cholesterol metabolism contribute to the pathological consequences of ApoE4 expression (TCW et al., 2022).

Recent studies have proposed a model by which astrocytes are able to buffer reactive oxygen species in the central nervous system as well as metabolically support neurons. Because neurons are vulnerable to reactive oxygen species damage from fatty acid oxidation (Schönfeld and Reiser, 2017), neuronal fatty acids are often taken up by surrounding astrocytes in ApoE-enriched particles and incorporated into lipid droplets (LDs), organelles that store neutral lipids within a phospholipid monolayer. LDs provide intracellular energy stores and often form in response to oxidative stress to prevent the toxic peroxidation of cellular lipids (Ralhan et al., 2021). The capacity of astrocytes to buffer neuronal fatty acids protects neurons from oxidative stress during periods of high activity (Chen et al., 2023), a state in which lipids are more prone to peroxidation, which is reinforced by the initiation of a transcriptional program in LD-accumulated astrocytes to detoxify and metabolize these lipids. In turn, astrocytic LDs can be mobilized through lipolysis and β-oxidation to provide fuel to sustain synaptic transmission (Ioannou et al., 2019; Figure 2D).

Defects in astrocytic lipid metabolism may also contribute to neurodegeneration. Lipid accumulation in the central nervous system has been associated with neurodegeneration since the first characterization of AD over a century ago (Stelzmann et al., 1995). Analysis of both postmortem human AD brains and mouse models has revealed that lipid accumulation is a pathological feature of AD and precedes Aβ aggregation and neurofibrillary tangle formation (Hamilton et al., 2015). The transfer of potentially toxic lipids from neurons to surrounding glia can be mediated by ApoE (Ioannou et al., 2019; Liu et al., 2017). The ApoE4 allele represents the strongest genetic risk factor for AD (Corder et al., 1993; Strittmatter et al., 1993) and is associated with hippocampal atrophy (Mishra et al., 2018; Shi et al., 2017). Astrocytes expressing ApoE4 exhibit various cellular dysfunctions, including disrupted fatty acid and sterol metabolism and efflux, triglyceride and reactive oxygen species accumulation, impaired mitochondrial function, reduced autophagic flux, impaired blood-brain barrier maintenance, and reduced support of hippocampal neurite growth (de Leeuw et al., 2022; Farmer et al., 2019; Jackson et al., 2022; Lee et al., 2023; Rawat et al., 2019; Sienski et al., 2021; Sun et al., 1998). Mechanistically, the pathological effects conferred by ApoE4 may be due, at least in part, to disrupted lipid transfer between neurons and glia (Ioannou et al., 2019; Liu et al., 2017; Qi et al., 2021), resulting in reduced neuronal lipid sequestration and astrocytic lipid utilization (Qi et al., 2021), as well as defective astrocytic cholesterol metabolism (TCW et al., 2022).

Overall, astrocytic glucose and lipid metabolism are critical for supporting the essential functions of the hippocampus. Glucose metabolism in the brain is profoundly reliant on astrocytic networks, which facilitate the neuronal activity-dependent flow of nutrients to support synaptic transmission. The sequestration of potentially toxic fatty acids and the efflux of cholesterol are critical mechanisms by which astrocytes support neuronal activity and have broad implications for the molecular basis of neurodegeneration.

## 5 Astrocytic encoding of neurotransmitters and gliotransmission

The metabolic and signaling functions of astrocytes are often complementary. For instance, the chemogenetic activation of CA1 astrocytes increases glucose metabolism specifically in the hippocampus (Ardanaz et al., 2024). Moreover, D-serine can be derived from glycolysis (Suzuki et al., 2015) and exhibits calcium-dependent release from astrocytes (Henneberger et al., 2010), thereby regulating neuronal activity. These essential astrocytic functions are closely interwoven by complex molecular crosstalk between astrocytes and neurons. Exploring the nature of this bidirectional communication is necessary to gain a unified view of astrocytic function and a comprehensive understanding of the hippocampus.

Astrocytes play a crucial role in supporting synaptic transmission by transporting and metabolizing neurotransmitters. These cells support both glutamatergic and GABAergic neuronal activity through the glutamate–glutamine cycle, a process essential for maintaining synaptic transmission. In this cycle, astrocytes produce glutamine from glucose, which is transported to neurons and converted into glutamate or GABA for presynaptic exocytosis. Following neurotransmission, glutamate and GABA are subsequently taken up by astrocytes from the synaptic cleft and metabolized to glutamine, allowing it to re-enter the cycle (Rothman et al., 2011). This process is necessary to maintain both excitatory (Tani et al., 2014) and inhibitory (Liang et al., 2006) synaptic transmission.

Beyond supporting neuronal activity through the metabolic flux of transmitters, astrocytes also express receptors for a variety of neurotransmitters and neuromodulators, including glutamate, GABA, acetylcholine, norepinephrine, adenosine triphosphate (ATP)/adenosine, and endocannabinoids. In turn, they are also able to release transmitters such as glutamate, GABA, ATP/adenosine, and D-serine through gliotransmission—the process by which astrocytes release transmitters to regulate synaptic activity (Araque et al., 2014)—to modulate synaptic transmission.

The specific expression of a dominant-negative form of soluble *N*-ethylmaleimide-sensitive factor attachment protein receptor (SNARE) in astrocytes to disrupt SNARE-mediated exocytosis—and thus gliotransmission—has demonstrated the importance of this process in maintaining hippocampal function. These studies have revealed the critical role that gliotransmission plays in astrocytic morphology (Viana et al., 2023), dendritic spine maturation (Sultan et al., 2015), hippocampal LTP (Abreu et al., 2023), neuronal coordination (Pascual et al., 2005; Sardinha et al., 2017), memory formation (Lee et al., 2014; Sardinha et al., 2017), and sleep homeostasis (Florian et al., 2011; Halassa et al., 2009). Here, we review some of the key transmitters encoded and released by astrocytes to facilitate the homeostatic and behavioral functions of the hippocampus.

### 5.1 Glutamate

Glutamate is the major excitatory neurotransmitter in the central nervous system. Hippocampal astrocytes express glutamate transporters (Huang et al., 2004) to modulate synaptic transmission through the electrogenic uptake of glutamate (Bergles and Jahr, 1997). This prevents excitotoxicity as well as neurotransmitter spillover, ensuring the specificity of synaptic transmission (Rothstein et al., 1996). Hippocampal astrocytes also express both ionotropic glutamate receptors (e.g., AMPARs, NMDARs, and kainic acid receptors) and metabotropic receptors, including mGluR1 and mGluR5 (Fan et al., 1999; Porter and McCarthy, 1995; Porter and McCarthy, 1996; Shelton and McCarthy, 1999). In the SC–CA1 region, astrocytic AMPAR activation inhibits potassium channels (Schröder et al., 2002) and facilitates the intercellular trafficking of glutamate across astrocytic networks to refine neuronal activity (Hösli et al., 2022; Rouach et al., 2008). NMDAR expression allows astrocytes to modulate presynaptic inputs to the CA1 (Chipman et al., 2021; Letellier et al., 2016). Furthermore, neuronal activity elicits astrocytic calcium activity through mGluR activation, which is required for the ensheathment of synapses by PAPs following LTP induction (Bernardinelli et al., 2014; Bowser and Khakh, 2004; Panatier et al., 2011; Porter and McCarthy, 1996; Rungta et al., 2016; Tang et al., 2015). Astrocytes are also capable of releasing glutamate downstream of calcium signaling as a gliotransmitter in response to neuronal activity. Recent evidence indicates that this ability to release glutamate is limited to a subset of astrocytes. In the hippocampus, these glutamate-releasing astrocytes are differentially distributed along the dorsoventral axis, with the highest proportion in the DG molecular layer, indicating regional diversity of gliotransmission (de Ceglia et al., 2023). This glutamate release can enhance LTP at SC–CA1 synapses (Adamsky et al., 2018; Perea and Araque, 2007). Furthermore, the specific deletion of vesicular glutamate transporter 1 in astrocytes diminishes contextual fear memory by disrupting glutamatergic gliotransmission in cortico-hippocampal circuitry (de Ceglia et al., 2023). These results suggest that glutamate release from astrocytes is sufficient to facilitate LTP in the hippocampus and that vesicular glutamate transporter 1-expressing astrocytes are specifically necessary for the consolidation of memories into extra-hippocampal areas.

### 5.2 GABA

GABA is the major inhibitory neurotransmitter in the central nervous system. GABAergic inputs impinge on nearly all excitatory synapses in the hippocampus. Astrocytes express both ionotropic (GABA_A_) and metabotropic (GABA_B_) receptors (Liu et al., 2022). GABA_B_ receptors allow these cells to respond to GABAergic neuronal activity to promote oscillatory hippocampal activity via glutamate release (Perea et al., 2016) or synaptic depression at SC–CA1 synapses via ATP/adenosine release (Serrano et al., 2006). Astrocytes are also capable of releasing GABA as a gliotransmitter (Gaidin et al., 2020; Le Meur et al., 2012) to diminish neuronal excitability (Gaidin et al., 2020). This process may be relevant in neurodegeneration, as multiple AD mouse models exhibit excessive GABA production and release from hippocampal astrocytes, which promotes tonic inhibition in the hippocampus through presynaptic GABA receptors, likely to compensate for synaptic hyperactivity. This GABA release impairs synaptic plasticity and memory formation, and suppressing this signaling restores memory deficits in these mice (Aldabbagh et al., 2022; Bhalla et al., 2025; Jo et al., 2014; Portal et al., 2024).

### 5.3 ATP/adenosine

ATP and its metabolite adenosine are neuromodulators closely linked to brain metabolism. Adenosine, the hydrolytic product of ATP, accumulates extracellularly during neuronal activity (Blutstein and Haydon, 2013; Dunwiddie et al., 1997). ATP released from SC–CA1 synapses drives astrocytic calcium signals through the activation of P2 purinergic receptors (Tang et al., 2015) as well as the cyclic AMP–protein kinase A signaling pathway (Theparambil et al., 2024). This latter pathway, downstream of adenosine A_2B_ receptor activation, elicits lactate release from astrocytes to facilitate neuronal activity and LTP. The knockdown of this receptor in astrocytes downregulates aerobic glycolysis and diminishes LTP induction, which is only partially rescued by lactate administration, indicating that the signaling pathway likely employs additional mechanisms beyond lactate release to promote LTP. This A_2B_ receptor activation in astrocytes is necessary for hippocampus-dependent memory formation and maintains sleep homeostasis by promoting delta-range neuronal activity during slow-wave sleep (Theparambil et al., 2024). This astrocytic encoding of purinergic neurotransmission may be critical in neurodegeneration, as APP/PS1 astrocytes aberrantly express adenosine A_2A_ receptors, impairing memory formation (Orr et al., 2015). ATP/adenosine also acts as a gliotransmitter to regulate both glutamatergic and GABAergic synaptic transmission. In the CA1 region, astrocytic ATP/adenosine release and presynaptic A_2A_ receptor activation modulate basal glutamate release (Panatier et al., 2011), while presynaptic A_1_ receptor activity facilitates the GABAergic induction of hetero-synaptic depression (Pascual et al., 2005; Serrano et al., 2006) and NMDAR-dependent spike timing-dependent LTD (Falcón-Moya et al., 2020; Pérez-Rodríguez et al., 2019). This signaling may be critical to maintaining sleep homeostasis, as ATP/adenosine release by astrocytes and its action on neuronal adenosine A_1_ receptors is necessary for both the accumulation of sleep pressure and the cognitive deficits associated with sleep deprivation in mice (Florian et al., 2011; Halassa et al., 2009).

### 5.4 D-serine

D-serine is a gliotransmitter and an NMDAR co-agonist; ligand binding to the NMDAR co-agonist site is critical for its function (Henneberger et al., 2010; Panatier et al., 2006; Papouin et al., 2012; Yang et al., 2003), as well as for LTP (Abreu et al., 2023; Henneberger et al., 2010; Yang et al., 2003) and LTD (Koh et al., 2022) induction. Studies in mice expressing a dominant-negative form of SNARE, which disrupts gliotransmitter release from astrocytes, have elucidated the significance of this astrocytic signaling. In the hippocampus, this abolishment leads to deficits in neuronal dendritic spine maturation (Sultan et al., 2015; Viana et al., 2023), diminished LTP at SC–CA1 synapses (Abreu et al., 2023), and disrupted coordination of cortico-hippocampal circuitry toward the formation of spatial memory (Sardinha et al., 2017). These effects are rescued by the administration of D-serine, which promotes the morphological development (Sultan et al., 2015; Viana et al., 2023) and integration (Sultan et al., 2015) of neuronal dendrites, reduces basal synaptic transmission to enhance LTP (Abreu et al., 2023), and promotes theta-phase oscillatory activity between the hippocampus and prefrontal cortex to facilitate spatial memory (Sardinha et al., 2017). These results suggest that D-serine, as a gliotransmitter, is essential for hippocampal functions from the molecular to the behavioral level. D-serine release can also be indirectly dependent on astrocytic glycolysis (Fernández-Moncada et al., 2024; Le Douce et al., 2020), a metabolic pathway likely relevant in neurodegeneration, as impaired glycolysis in AD mouse models leads to a reduction in D-serine levels concomitant with spatial memory deficits, which are rescued by D-serine administration (Le Douce et al., 2020).

### 5.5 Acetylcholine

Acetylcholine is a neuromodulator crucial for the acquisition of hippocampus-dependent memory. Cholinergic afferents from the basal forebrain form part of the reticular activating system, modulating arousal and acting as a neurochemical correlate of wakefulness and vigilance (Zaborszky et al., 2008). Acetylcholine can elicit calcium activity in hippocampal astrocytes through binding nicotinic (Sharma and Vijayaraghavan, 2001) or muscarinic (Araque et al., 2002; Navarrete et al., 2012) cholinergic receptors. This signaling from the basal forebrain through hippocampal astrocytes can induce LTP at SC–CA1 synapses, dependent on presynaptic mGluR activity (Navarrete et al., 2012). Studies have elucidated the behavioral consequences of this signaling, suggesting that D-serine acts as the downstream mechanism through which cholinergic afferents mediate hippocampal function through astrocytes (Ma et al., 2023; Pabst et al., 2016; Papouin et al., 2017). Septal cholinergic signaling elicits nicotinic cholinergic receptor activation and calcium-dependent D-serine release from hippocampal astrocytes to regulate NMDAR activation, which activates hilar interneurons in the perforant pathway–DG to inhibit dentate gyrus granule cells (Pabst et al., 2016). In the direct pathway–CA1, this signaling promotes the formation of temporally-associated memory (Ma et al., 2023; Figure 1E). At SC–CA1 synapses, this cholinergic-mediated astrocytic activity modulates hippocampus-dependent memory formation across the circadian cycle (Papouin et al., 2017).

### 5.6 Norepinephrine

Norepinephrine is a catecholamine primarily released by the locus coeruleus and modulates the vigilance state of the animal (Levitt and Moore, 1978). Projections from the locus coeruleus reach many areas of the brain, including the hippocampus. Hippocampal astrocytes respond to norepinephrine with calcium transients (Duffy and MacVicar, 1995) through their expression of β2-adrenergic receptors (Gao et al., 2016) and α1-adrenergic receptors (Duffy and MacVicar, 1995). The binding of norepinephrine to β2-adrenergic receptors allows astrocytes to respond to noradrenergic activity with the release of lactate to support neurons during hippocampus-dependent memory formation (Gao et al., 2016). Astrocytes thus regulate synaptic activity according to vigilance state and mediate the noradrenergic modulation of hippocampal function (Gao et al., 2016; Koh et al., 2022). This mechanism highlights the close relationship between the signaling and metabolic functions of astrocytes in facilitating neuronal activity during memory formation. In addition, astrocytic α1-adrenergic receptor activation elicits calcium activity and ATP/adenosine release at SC–CA1 synapses, which suppresses synaptic activity through the activation of presynaptic adenosine A_1_ receptors (Lefton et al., 2025), suggesting that noradrenergic modulation of hippocampal synapses is mediated by astrocytic purinergic signaling. Astrocytes may also play a role in integrating noradrenergic afferents to the hippocampus. Noradrenergic projections from the locus coeruleus activate α1-adrenergic receptors on astrocytes to elicit global astrocytic calcium activity that propagates toward the soma as a mouse approaches a reward. This calcium activity likely reflects past events, such as changes in neuronal activity, locomotion, and pupil dilation stimulated by noradrenergic activity. Furthermore, this somatic calcium activity occurs across a longer timescale than the neuronal activity it may encode (Rupprecht et al., 2024). The implications of this encoding remain obscure, but it positions hippocampal astrocytes as slow integrators of noradrenergic afferents.

### 5.7 Endocannabinoids

Endocannabinoids are a class of neuromodulators that mediate synaptic plasticity in the hippocampus (Izumi and Zorumski, 2012; Ohno-Shosaku et al., 2007; Zhu and Lovinger, 2007). The expression of cannabinoid receptor 1 (CB_1_R) in astrocytes is necessary to maintain synchronized neuronal activity in the hippocampus during epileptiform events (Coiret et al., 2012), indicating that this signaling underlies a mechanism by which astrocytes coordinate neuronal circuits. Furthermore, the activation of astrocytic CB_1_Rs also upregulates lactate metabolism, thereby promoting D-serine synthesis. Mice lacking astrocytic CB_1_R exhibit impaired hippocampus-dependent memory, which is rescued by D-serine administration or upregulation of the glycolytic pathway producing this gliotransmitter (Fernández-Moncada et al., 2024). Dendritic spiking—an action potential generated in a dendrite—of CA1 pyramidal neurons is critical in encoding spatial information. This activity is elicited by theta-range pyramidal neuronal activity in the CA1 region, the co-occurrence of which is critical for the formation of spatial memory (Epsztein et al., 2011; Harvey et al., 2009). Pyramidal cell activity in the theta range occurs during spatial exploration and activates CB_1_Rs on astrocytes, which exhibit calcium transients and release D-serine to lower the threshold of dendritic spiking; this astrocytic signaling is necessary for the formation of spatial memory (Bohmbach et al., 2022). In this manner, astrocytes may provide the mechanistic link between theta-range activity in pyramidal cells and the potentiation of dendritic spiking to facilitate the formation of spatial memory.

## 6 Discussion

To fully understand hippocampal function, it is increasingly apparent that astrocytes must be considered for their role in the active encoding, integration, and modulation of information across synapses in this region. Here, we have summarized the circuit-specific roles of astrocytes, which depend both on the fine-tuning of single synapses in their cellular domains and on the coordination and metabolic support of neuronal circuits through astrocytic networks. These dual perspectives are intricately coordinated by complex molecular crosstalk between astrocytes and neurons through the processes of neurotransmission and gliotransmission.

Hippocampal astrocytes respond dynamically to the spatiotemporal encoding of experience by hippocampal neurons, the energetic needs of local circuitry, and the broader contexts of brain state, such as vigilance state, to shape behavioral outcomes. These processes converge on the precise, plastic control of synaptic transmission to support the functions of the hippocampus as a scaffold for memory, underpinning synaptic plasticity and memory formation in this region, while also facilitating cortico-hippocampal communication to mediate the long-term consolidation and generalization of memory.

Understanding the roles of astrocytes in the acquisition and consolidation of hippocampus-dependent memory is expected to deepen our understanding of the behavioral functions of this brain region. Determining the implications of the diverse array of cellular signaling and its integration with metabolism, both centrally and peripherally, in hippocampal astrocytes will be key to elucidating how these cells encode and modulate the flow of information across synapses. This aim may yield invaluable insights into the cellular mechanisms of memory formation.

Overall, astrocytes are indispensable for hippocampal function, both locally and globally. We hope that the conceptual framework of their circuit-dependent roles provided here will foster clarity for future research, wherein a more comprehensive understanding of the hippocampus may be achieved through investigations into the dynamics of astrocytic activity in this brain area. For instance, identifying the effectors downstream of calcium signaling that facilitate sleep homeostasis and memory formation, clarifying the implications of cellular signaling beyond calcium, defining the precise role of neuron–astrocyte metabolic coupling in learning and memory, and establishing whether astrocytes contribute to the allocation of sparse neuronal populations to an engram all represent frontier areas. Extending the lines of investigation outlined in this review holds promise for advancing a deeper understanding of the behavioral functions of the hippocampus.
